# 2-{[(2-Methyl­prop-2-en-1-yl)­oxy]meth­yl}-6-phenyl-2,3,4,5-tetra­hydro-1,2,4-triazine-3,5-dione

**DOI:** 10.1107/S160053681105567X

**Published:** 2012-01-11

**Authors:** Nasser R. El-Brollosy, Ali A. El-Emam, Abdulghafoor A. Al-Turkistani, Seik Weng Ng

**Affiliations:** a Department of Pharmaceutical Chemistry, College of Pharmacy, King Saud University, Riyadh 11451, Saudi Arabia; b Department of Chemistry, University of Malaya, 50603 Kuala Lumpur, Malaysia; c Chemistry Department, Faculty of Science, King Abdulaziz University, PO Box 80203 Jeddah, Saudi Arabia

## Abstract

The 1,2,4-triazine ring in the title compound, C_14_H_15_N_3_O_3_, is approximately planar (r.m.s. deviation = 0.019 Å); the C atom at the 6-position deviates by 0.026 (2) Å from the mean plane whereas the C atom at the 2-position deviates by 0.166 (4) Å in the opposite direction. The triazine ring is oriented at 8.60 (13)° with respect to the phenyl ring. The imino group is hydrogen-bond donor to the exocyclic O atom at the 3-position of an adjacent mol­ecule, the hydrogen bond generating an inversion dimer.

## Related literature

For the synthesis and anti­microbial activity of the title compound, see: El-Brollosy (2008 ▶).
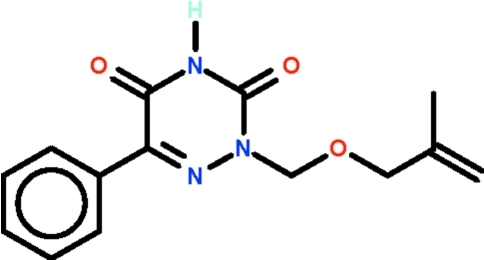


## Experimental

### 

#### Crystal data

C_14_H_15_N_3_O_3_
*M* *_r_* = 273.29
Triclinic, 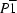

*a* = 4.6162 (5) Å
*b* = 11.7896 (14) Å
*c* = 12.5769 (10) Å
α = 81.588 (8)°
β = 85.836 (7)°
γ = 87.245 (9)°
*V* = 674.84 (12) Å^3^
*Z* = 2
Mo *K*α radiation
μ = 0.10 mm^−1^
*T* = 100 K
0.30 × 0.03 × 0.03 mm

#### Data collection

Agilent SuperNova Dual diffractometer with an Atlas detector
Absorption correction: multi-scan (*CrysAlis PRO*; Agilent, 2010 ▶) *T*_min_ = 0.972, *T*_max_ = 0.997
9875 measured reflections
3088 independent reflections
1878 reflections with *I* > 2σ(*I*)
*R*_int_ = 0.086

#### Refinement

*R*[*F* ^2^ > 2σ(*F* ^2^)] = 0.081
*wR*(*F* ^2^) = 0.250
*S* = 1.01
3088 reflections
186 parameters
1 restraint
H atoms treated by a mixture of independent and constrained refinement
Δρ_max_ = 0.53 e Å^−3^
Δρ_min_ = −0.45 e Å^−3^

### 

Data collection: *CrysAlis PRO* (Agilent, 2010 ▶); cell refinement: *CrysAlis PRO*; data reduction: *CrysAlis PRO*; program(s) used to solve structure: *SHELXS97* (Sheldrick, 2008 ▶); program(s) used to refine structure: *SHELXL97* (Sheldrick, 2008 ▶); molecular graphics: *X-SEED* (Barbour, 2001 ▶); software used to prepare material for publication: *publCIF* (Westrip, 2010 ▶).

## Supplementary Material

Crystal structure: contains datablock(s) global, I. DOI: 10.1107/S160053681105567X/xu5422sup1.cif

Structure factors: contains datablock(s) I. DOI: 10.1107/S160053681105567X/xu5422Isup2.hkl

Supplementary material file. DOI: 10.1107/S160053681105567X/xu5422Isup3.cml

Additional supplementary materials:  crystallographic information; 3D view; checkCIF report

## Figures and Tables

**Table 1:** Hydrogen-bond geometry (Å, °)

*D*—H⋯*A*	*D*—H	H⋯*A*	*D*⋯*A*	*D*—H⋯*A*
N1—H1⋯O1^i^	0.89 (1)	1.93 (1)	2.802 (3)	168 (4)

Symmetry code: (i) 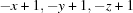.

**Fig. 1.**
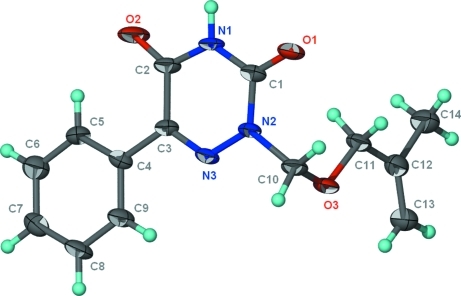
Thermal ellipsoid plot (Barbour, 2001) of C14H15N3O3 at the 70% probability level; hydrogen atoms are drawn as spheres of arbitrary radius.

**Fig. 2.**
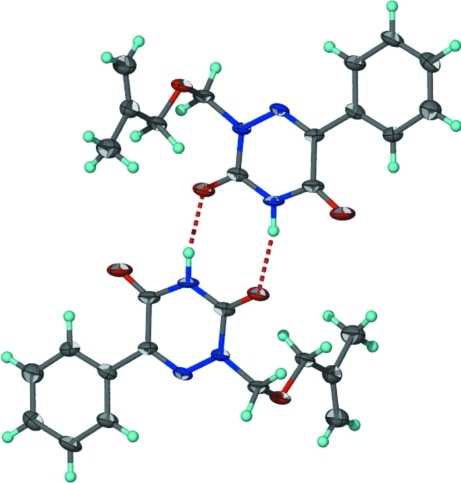
Dimeric hydrogen-bonded structure.

## supplementary crystallographic information

### Comment

The compound (Scheme I) was synthesized for an evaluation of its antimicrobial activity (El-Brollosy, 2008). The 1,2,4-triazine ring of is planar (r.m.s. deviation 0.019 Å); the C atom at the 6-position deviates by 0.026 (2) Å from the mean plane whereas the C atom at the 2-position deviates from the mean plane by 0.166 (4) Å in the opposite direction (Fig. 1). The amino group is hydrogen-bond donor to the exocyclic O atom at the 3-position, the hydrogen bond generating a centrosymmetric dimer (Table 1, Fig. 2).

### Experimental

The compound was synthesized by using a reported method (El-Brollosy, 2008), and was recrystallized from ethanol.

### Refinement

Carbon-bound H-atoms were placed in calculated positions [C–H 0.95 to 0.98 Å, *U*_iso_(H) 1.2 to 1.5*U*_eq_(C)] and were included in the refinement in the riding model approximation.

The imino H-atom was located in a difference Fourier map, and was refined with a distance restraint of N–H 0.88±0.01 Å; its temperature factor was refined.

### Crystal data

**Table:**

C_14_H_15_N_3_O_3_	*Z* = 2
*M**_r_* = 273.29	*F*(000) = 288
Triclinic, *P*1	*D*_x_ = 1.345 Mg m^−^^3^
Hall symbol: -P 1	Mo *K*α radiation, λ = 0.71073 Å
*a* = 4.6162 (5) Å	Cell parameters from 1917 reflections
*b* = 11.7896 (14) Å	θ = 2.6–27.5°
*c* = 12.5769 (10) Å	µ = 0.10 mm^−^^1^
α = 81.588 (8)°	*T* = 100 K
β = 85.836 (7)°	Prism, colorless
γ = 87.245 (9)°	0.30 × 0.03 × 0.03 mm
*V* = 674.84 (12) Å^3^	

### Data collection

**Table:**

Agilent SuperNova Dual diffractometer with an Atlas detector	3088 independent reflections
Radiation source: SuperNova (Mo) X-ray Source	1878 reflections with *I* > 2σ(*I*)
Mirror	*R*_int_ = 0.086
Detector resolution: 10.4041 pixels mm^-1^	θ_max_ = 27.6°, θ_min_ = 2.6°
ω scan	*h* = −6→6
Absorption correction: multi-scan (CrysAlis PRO; Agilent, 2010)	*k* = −15→15
*T*_min_ = 0.972, *T*_max_ = 0.997	*l* = −16→16
9875 measured reflections	

### Refinement

**Table:**

Refinement on *F*^2^	Primary atom site location: structure-invariant direct methods
Least-squares matrix: full	Secondary atom site location: difference Fourier map
*R*[*F*^2^ > 2σ(*F*^2^)] = 0.081	Hydrogen site location: inferred from neighbouring sites
*wR*(*F*^2^) = 0.250	H atoms treated by a mixture of independent and constrained refinement
*S* = 1.01	*w* = 1/[σ^2^(*F*_o_^2^) + (0.1384*P*)^2^] where *P* = (*F*_o_^2^ + 2*F*_c_^2^)/3
3088 reflections	(Δ/σ)_max_ = 0.001
186 parameters	Δρ_max_ = 0.53 e Å^−^^3^
1 restraint	Δρ_min_ = −0.45 e Å^−^^3^

### Fractional atomic coordinates and isotropic or equivalent isotropic displacement parameters (Å^2^)

**Table:**

	*x*	*y*	*z*	*U*_iso_*/*U*_eq_	
O1	0.5220 (4)	0.40726 (17)	0.61847 (14)	0.0268 (5)	
O2	−0.1301 (4)	0.7060 (2)	0.55343 (15)	0.0370 (6)	
O3	0.0363 (4)	0.24966 (17)	0.82369 (14)	0.0261 (5)	
N1	0.1946 (5)	0.5578 (2)	0.58770 (16)	0.0224 (6)	
H1	0.277 (7)	0.580 (3)	0.5224 (14)	0.062 (12)*	
N2	0.1685 (5)	0.4378 (2)	0.74966 (16)	0.0219 (5)	
N3	−0.0574 (4)	0.5017 (2)	0.78892 (16)	0.0216 (5)	
C1	0.3099 (6)	0.4641 (2)	0.6493 (2)	0.0227 (6)	
C2	−0.0396 (6)	0.6266 (3)	0.6174 (2)	0.0248 (6)	
C3	−0.1586 (5)	0.5929 (2)	0.73057 (19)	0.0207 (6)	
C4	−0.3984 (5)	0.6607 (2)	0.7819 (2)	0.0217 (6)	
C5	−0.5471 (6)	0.7527 (2)	0.7267 (2)	0.0257 (6)	
H5	−0.5003	0.7749	0.6520	0.031*	
C6	−0.7664 (6)	0.8135 (3)	0.7801 (2)	0.0305 (7)	
H6	−0.8691	0.8762	0.7415	0.037*	
C7	−0.8330 (6)	0.7822 (3)	0.8889 (2)	0.0292 (7)	
H7	−0.9804	0.8239	0.9252	0.035*	
C8	−0.6862 (6)	0.6905 (3)	0.9448 (2)	0.0299 (7)	
H8	−0.7345	0.6689	1.0195	0.036*	
C9	−0.4675 (6)	0.6294 (3)	0.8926 (2)	0.0263 (7)	
H9	−0.3655	0.5668	0.9318	0.032*	
C10	0.2431 (6)	0.3324 (3)	0.8212 (2)	0.0248 (6)	
H10A	0.4350	0.3013	0.7963	0.030*	
H10B	0.2581	0.3506	0.8950	0.030*	
C11	0.0328 (6)	0.2037 (3)	0.7240 (2)	0.0266 (7)	
H11A	0.2318	0.1769	0.7022	0.032*	
H11B	−0.0311	0.2649	0.6670	0.032*	
C12	−0.1666 (6)	0.1064 (3)	0.7350 (2)	0.0297 (7)	
C13	−0.2911 (7)	0.0595 (3)	0.8283 (2)	0.0357 (8)	
H13A	−0.2548	0.0879	0.8928	0.043*	
H13B	−0.4166	−0.0025	0.8307	0.043*	
C14	−0.2130 (7)	0.0672 (3)	0.6292 (2)	0.0375 (8)	
H14A	−0.3299	−0.0013	0.6420	0.056*	
H14B	−0.0245	0.0489	0.5934	0.056*	
H14C	−0.3148	0.1284	0.5833	0.056*	

### Atomic displacement parameters (Å^2^)

**Table:**

	*U*^11^	*U*^22^	*U*^33^	*U*^12^	*U*^13^	*U*^23^
O1	0.0280 (11)	0.0367 (12)	0.0136 (9)	0.0045 (9)	0.0047 (8)	−0.0017 (8)
O2	0.0420 (12)	0.0492 (15)	0.0141 (10)	0.0119 (11)	0.0064 (8)	0.0055 (9)
O3	0.0328 (11)	0.0321 (12)	0.0121 (9)	0.0010 (9)	0.0050 (7)	−0.0025 (8)
N1	0.0264 (12)	0.0306 (14)	0.0083 (10)	−0.0017 (10)	0.0040 (9)	0.0015 (10)
N2	0.0275 (12)	0.0259 (13)	0.0108 (10)	0.0025 (10)	0.0039 (9)	−0.0007 (9)
N3	0.0249 (12)	0.0287 (13)	0.0108 (10)	0.0010 (10)	0.0027 (8)	−0.0042 (9)
C1	0.0241 (14)	0.0313 (16)	0.0126 (12)	−0.0017 (12)	0.0007 (10)	−0.0040 (11)
C2	0.0284 (14)	0.0340 (17)	0.0106 (12)	0.0002 (12)	0.0005 (10)	0.0000 (11)
C3	0.0235 (14)	0.0255 (15)	0.0122 (12)	−0.0018 (11)	0.0011 (10)	−0.0003 (10)
C4	0.0203 (13)	0.0283 (16)	0.0165 (12)	−0.0005 (11)	0.0025 (10)	−0.0058 (11)
C5	0.0323 (15)	0.0263 (15)	0.0168 (13)	−0.0006 (12)	0.0022 (11)	0.0006 (11)
C6	0.0307 (16)	0.0345 (18)	0.0260 (15)	0.0010 (14)	−0.0010 (12)	−0.0046 (13)
C7	0.0299 (15)	0.0323 (17)	0.0266 (14)	−0.0006 (13)	0.0038 (12)	−0.0107 (13)
C8	0.0314 (16)	0.0422 (19)	0.0161 (13)	0.0044 (14)	0.0045 (11)	−0.0086 (13)
C9	0.0257 (14)	0.0360 (17)	0.0155 (13)	0.0053 (13)	0.0019 (10)	−0.0024 (12)
C10	0.0289 (14)	0.0339 (17)	0.0104 (11)	0.0030 (12)	−0.0002 (10)	−0.0009 (11)
C11	0.0333 (15)	0.0326 (17)	0.0128 (12)	0.0060 (13)	0.0015 (11)	−0.0037 (12)
C12	0.0353 (16)	0.0298 (17)	0.0223 (14)	0.0072 (13)	0.0012 (12)	−0.0024 (12)
C13	0.0461 (18)	0.0342 (18)	0.0258 (15)	−0.0044 (15)	0.0058 (13)	−0.0038 (13)
C14	0.0500 (19)	0.0383 (19)	0.0240 (15)	−0.0034 (16)	0.0003 (13)	−0.0045 (14)

### Geometric parameters (Å, °)

**Table:**

O1—C1	1.230 (3)	C6—H6	0.9500
O2—C2	1.220 (3)	C7—C8	1.378 (4)
O3—C10	1.393 (3)	C7—H7	0.9500
O3—C11	1.438 (3)	C8—C9	1.393 (4)
N1—C1	1.362 (4)	C8—H8	0.9500
N1—C2	1.383 (4)	C9—H9	0.9500
N1—H1	0.888 (10)	C10—H10A	0.9900
N2—N3	1.362 (3)	C10—H10B	0.9900
N2—C1	1.379 (3)	C11—C12	1.490 (4)
N2—C10	1.466 (3)	C11—H11A	0.9900
N3—C3	1.297 (4)	C11—H11B	0.9900
C2—C3	1.493 (3)	C12—C13	1.325 (4)
C3—C4	1.498 (3)	C12—C14	1.503 (4)
C4—C5	1.381 (4)	C13—H13A	0.9500
C4—C9	1.405 (4)	C13—H13B	0.9500
C5—C6	1.401 (4)	C14—H14A	0.9800
C5—H5	0.9500	C14—H14B	0.9800
C6—C7	1.380 (4)	C14—H14C	0.9800
			
C10—O3—C11	113.35 (19)	C7—C8—H8	119.7
C1—N1—C2	126.5 (2)	C9—C8—H8	119.7
C1—N1—H1	119 (3)	C8—C9—C4	119.7 (3)
C2—N1—H1	115 (3)	C8—C9—H9	120.1
N3—N2—C1	124.4 (2)	C4—C9—H9	120.1
N3—N2—C10	114.3 (2)	O3—C10—N2	111.8 (2)
C1—N2—C10	121.2 (2)	O3—C10—H10A	109.3
C3—N3—N2	120.6 (2)	N2—C10—H10A	109.3
O1—C1—N1	123.2 (2)	O3—C10—H10B	109.3
O1—C1—N2	122.5 (3)	N2—C10—H10B	109.3
N1—C1—N2	114.2 (2)	H10A—C10—H10B	107.9
O2—C2—N1	120.4 (2)	O3—C11—C12	111.1 (2)
O2—C2—C3	126.1 (3)	O3—C11—H11A	109.4
N1—C2—C3	113.5 (2)	C12—C11—H11A	109.4
N3—C3—C2	120.7 (2)	O3—C11—H11B	109.4
N3—C3—C4	117.0 (2)	C12—C11—H11B	109.4
C2—C3—C4	122.4 (3)	H11A—C11—H11B	108.0
C5—C4—C9	119.2 (2)	C13—C12—C11	123.4 (3)
C5—C4—C3	123.4 (2)	C13—C12—C14	123.5 (3)
C9—C4—C3	117.3 (2)	C11—C12—C14	113.1 (2)
C4—C5—C6	120.5 (2)	C12—C13—H13A	120.0
C4—C5—H5	119.8	C12—C13—H13B	120.0
C6—C5—H5	119.8	H13A—C13—H13B	120.0
C7—C6—C5	119.9 (3)	C12—C14—H14A	109.5
C7—C6—H6	120.0	C12—C14—H14B	109.5
C5—C6—H6	120.0	H14A—C14—H14B	109.5
C8—C7—C6	120.1 (3)	C12—C14—H14C	109.5
C8—C7—H7	119.9	H14A—C14—H14C	109.5
C6—C7—H7	119.9	H14B—C14—H14C	109.5
C7—C8—C9	120.5 (3)		
			
C1—N2—N3—C3	1.5 (4)	C2—C3—C4—C5	6.4 (4)
C10—N2—N3—C3	−175.2 (2)	N3—C3—C4—C9	8.3 (4)
C2—N1—C1—O1	−178.9 (2)	C2—C3—C4—C9	−172.1 (2)
C2—N1—C1—N2	1.1 (4)	C9—C4—C5—C6	−0.6 (4)
N3—N2—C1—O1	176.5 (2)	C3—C4—C5—C6	−179.1 (2)
C10—N2—C1—O1	−7.1 (4)	C4—C5—C6—C7	0.6 (4)
N3—N2—C1—N1	−3.5 (4)	C5—C6—C7—C8	−0.6 (4)
C10—N2—C1—N1	172.9 (2)	C6—C7—C8—C9	0.7 (4)
C1—N1—C2—O2	−177.2 (2)	C7—C8—C9—C4	−0.7 (4)
C1—N1—C2—C3	2.8 (4)	C5—C4—C9—C8	0.7 (4)
N2—N3—C3—C2	3.0 (4)	C3—C4—C9—C8	179.2 (2)
N2—N3—C3—C4	−177.40 (19)	C11—O3—C10—N2	68.7 (3)
O2—C2—C3—N3	175.1 (3)	N3—N2—C10—O3	72.8 (2)
N1—C2—C3—N3	−4.9 (4)	C1—N2—C10—O3	−104.0 (3)
O2—C2—C3—C4	−4.5 (4)	C10—O3—C11—C12	173.3 (2)
N1—C2—C3—C4	175.5 (2)	O3—C11—C12—C13	−8.5 (4)
N3—C3—C4—C5	−173.3 (2)	O3—C11—C12—C14	171.2 (2)

### Hydrogen-bond geometry (Å, °)

**Table:**

*D*—H···*A*	*D*—H	H···*A*	*D*···*A*	*D*—H···*A*
N1—H1···O1^i^	0.89 (1)	1.93 (1)	2.802 (3)	168 (4)

Symmetry codes: (i) −*x*+1, −*y*+1, −*z*+1.
